# Feasibility of screening for cognitive impairment among older persons and referral by community health workers in Wakiso district, Uganda

**DOI:** 10.1186/s12888-023-05015-0

**Published:** 2023-07-24

**Authors:** Racheal Alinaitwe, Seggane Musisi, David Mukunya, Yvette Wibabara, Byamah B Mutamba, Noeline Nakasujja

**Affiliations:** 1 Department of Psychiatry, Makerere University College of Health Sciences, Kampala, Uganda; 2 Department of Community and Public Health, Busitema University, Mbale, Uganda; 3 Clinical Epidemiology Unit, Makerere University, Kampala, Uganda; 4 Butabika National Referral Mental Hospital, Kampala, Uganda

**Keywords:** Cognitive impairment, Older persons, Community health workers, Referral

## Abstract

**Background:**

In Uganda, cognitive impairment in older persons aged ≥ 60 years is often undiagnosed due to inadequate appreciation of the condition compounded with limitations of trained human resource able to conduct appropriate cognitive evaluations. Use of Community Health Workers (CHWs) especially in hard-to-reach communities can be an important link for older persons to the health facilities where they can receive adequate evaluations and interventions for cognitive challenges. The aim of the study was to assess the feasibility of screening for cognitive impairment among older persons and referral by CHWs in Wakiso district, Uganda.

**Methods:**

This was a sequential explanatory mixed methods study. The CHWs received a one-day training on causes, signs and symptoms, and management of cognitive impairment and screened older persons ≥ 60 years for cognitive impairment using the Alzheimer’s Disease scale 8 (AD8). Psychiatric clinical officers (PCOs) administered the AD8 to the older persons after assessment by the CHWs who then referred them for appropriate clinical care. We conducted Kappa statistic for agreement between the CHWs and PCOs and compared raw scores of the CHWs to Experts scores using Bland Altman and pair plots and corresponding analyses. We also conducted focus group discussions for the older persons, caregivers and CHWs.

**Results:**

We collected data from 385 older persons. We involved 12 CHWs and 75% were females, majority were married (58.3%) with at least a secondary education (66.7%). There was 96.4% (CI 94.5–98.2%) agreement between PCOs and CHWs in identifying cognitive impairment with the PCOs identifying 54/385 (14.0: 95%CI 10.7–17.9%) older persons compared to 58/385 (15.1: 95%CI 11.6–19.0%) identified by CHWs. Of the 58 identified to have cognitive impairment by the CHWs, 93.1% were referred for care. The average difference between the score of the expert and that of the CHW was − 0.042 with a 95% CI of -1.335 to 1.252. Corresponding Bland Altman and pair plots showed high agreement between the measurements although CHWs scored higher values with increasing scores.

**Conclusion:**

CHWs can be trained to identify and refer older persons with cognitive impairment in the communities.

**Supplementary Information:**

The online version contains supplementary material available at 10.1186/s12888-023-05015-0.

## Background

The population of older persons (≥ 60 years) is increasing in Low and Middle Income countries with a projected increment of 2.9% annually before 2050 [1]. Currently 11% of the world’s population is above 60 years with 5% of the population in sub-Saharan Africa being aged ≥ 60 years [1, 2]. In Uganda, the older persons account for 1.6 million (5%) of the population [3].

Statistics about psychiatric disorders among the elderly in the general population are generally scanty. A study done on medical and surgical wards of Mulago National Referral Hospital found cognitive impairment to be one of the most common psychiatric disorders among the older individuals at a prevalence of 24.4% [4]. In another study in Western Uganda, Atim et al. (2021) found a 28.1% prevalence of neurocognitive impairment in hospitalised older persons aged ≥ 60 years of age [5]. However, cognitive impairment is often missed or misdiagnosed by non-mental health practitioners.

The health professionals commonly involved in cognitive assessments for older persons are mostly stationed at urban healthcare facilities. These cadres include nurses, general practitioners, psychiatric clinical officers (PCOs), psychiatrists and psychologists [6]. However, many of the older persons have difficulties accessing these mental health facilities due to physical illnesses and disability [7], long distances to the health facilities, long lines at the health facilities and lack of community awareness about the mental health needs of the older persons [8].

As a result of limited financial and human resources for mental health, community-based care is becoming a preferred mode of care for people with mental illness especially in hard to reach communities so as to bridge the gap in accessing care [9]. This care involves use of community health workers (CHWs) of Village Health Teams (VHTs) that operate within the indigenous communities where they live hence are known and easily accepted by the community [10]. The CHWs act as a link between the health service users and the health care providers for hard to reach rural communities [11]. CHWs are the first port of call in the health care system in Uganda because they work closely with the communities to identify and manage minor ailments. For complicated cases, CHWs write referrals for the patients to be taken to appropriate health facilities. The referral notes have a feedback form that the patient brings back to the CHWs and the CHWs also follow up with the patients at the health facility to ensure care was sought [12]. However, in Uganda the current training of CHWs involves only basic health topics of malaria, sanitation and hygiene, pneumonia, tuberculosis and HIV prevention among children and pregnant women with no mental health component [13, 14]. Older persons are an increasing population in our communities with mental health issues like cognitive impairment yet they cannot easily access the health care facilities for care hence poor health outcomes [3, 4]. Involving the CHWs in identifying and referring older persons with cognitive impairment could lead to early treatment in this population with possibly better treatment out comes and an improved quality of life. We aimed to assess the feasibility of involving CHWs in identifying and referring older persons with cognitive impairment for care in Wakiso district, Uganda.

## Methods

The aim of the study was to assess the feasibility of involving CHWs in identifying and referring older persons with cognitive impairment for care in Wakiso district, Uganda.

We conducted a sequential explanatory mixed methods study in Nangabo division of Wakiso district Uganda, chosen because of its mix of urban and rural communities. Wakiso is located in the South-central region of Uganda, 12 km from the capital city and has a population of 1,997,418 with 2.3% (45,077) aged ≥ 60 years. Nangabo division has 52 villages and each village has four Community Health Workers (two of the four are specifically in-charge of drug administration and the other two in-charge of mobilisation). We chose a mixed methods study because we needed the qualitative findings to further explain the quantitative findings on feasibility.

We included older persons aged ≥ 60 years who had lived in Nangabo for at least 6 months in 12 villages comprising the catchment area for Kasangati Health Centre IV. We utilised one of the CHWs in charge of mobilisation (these are in constant contact with the health centre) per village aged ≥ 18 years hence a total of 12 CHWs. Principal investigator (PI) with the help of the lead CHW selected the CHWs who met inclusion criteria and these were explained the purpose of the study. Written informed consent and their socio-demographic information were obtained from the CHWs by the Research assistants (RAs) who were psychiatric clinical officers (PCOs) with diploma level mental health training hence were considered as experts. We obtained informed consent from the CHWs because they were considered as participants in this study and we had to collect sociodemographic data from them. The PCOs had received training in responsible conduct of research and good clinical practice.

The CHWs were trained by the principal investigator (PI) on cognitive impairment, its assessment and management. The CHWs also received a training by the PCOs and principal investigator (PI) on the administration and scoring of the AD8 for half a day including role-plays. We conducted six role plays involving administration of the AD8.

Data were collected over a period of two months from December 2020 to February 2021.

### Study procedure

With guidance from the CHWs, older people’s homes were conveniently consecutively sampled followed by the PI or the PCOs assessing for study eligibility of the older persons (and their caregivers). The purpose of the study was then explained to the older persons and their caregivers who met the inclusion criteria. Capacity to consent for each older person was assessed by the University of California, San Diego Brief Assessment of Capacity to Consent (UBACC) and written informed consent was sought from older persons. For older persons who were not mentally competent to give consent (as assessed by the UBACC ≤ 14 after 3 attempts), their key relative/caretaker would provide the alternative consent. Socio-demographic questionnaires were administered to the older persons and the CHWs by the PCOs. The CHWs administered the AD8 to the older persons or informants of the older person to screen for cognitive impairment. Individuals who were found to have AD8 scores ≥ 2 were to be referred by writing a referral note to the PCOs or PI. The PCOs and PI also repeated the administration of the AD8 to same population of older persons screened by the CHWs on the same day regardless of their score on the AD8. The PCOs were trained in qualitative data collection to conduct six Focus Group Discussions (FGDs) (two for CHWs, two for older persons and two for caregivers) with each FGD having 6–8 people. The sample size of 6 FGDs was based on the literature that states that at least 4 FGDs are sufficient to identify a range of new issues and 2 FGDs per stratum are needed for saturation [15]. We developed separate interview guides for the different categories of study participants for the different FGDs. The guides were used flexibly and modified according to the preliminary findings and as need arose in the course of the study. The interviews lasted between 20 and 80 min and were conducted in Luganda and English languages by the PIs. Participants provided written informed consent to participate in the FGDs. One PCO was a note taker in each focus group discussion (FGDs). Participants were briefed on the main purpose of the discussion and emphasis was placed on inter participant discussions and confidentiality. All audio files were be transcribed and translated into the English language then analysed using thematic approach.

### Measurements, tools and data collection

#### Socio-demographic questionnaire

This was administered by the PI or PCOs to CHWs and the older persons (after written informed consent was obtained) to collect data on age, gender, marital status, religion, tribe, education attained, employment/income source and physical illness.

#### The Alzheimer’s disease-8 (AD8)

The AD8 is a brief, sensitive measure that validly and reliably differentiates between individuals with dementia and those without [16]. It can be used as a general screening device to detect cognitive change regardless of etiology and with different types of informants [16]. It has been used in detecting cognitive impairment in a community setting and was found to have superior sensitivity compared to the short form of the informant questionnaire on cognitive decline in the elderly (IQCODE) [17].

#### Topic guides

These were drafted by the PI with the guidance of a qualitative data expert who was to do the analysis. The guides were translated by a linguistic scholar into Luganda which is the commonly spoken local language. The topic guides looked at ease of administration of the AD-8 by the CHWs, interaction of the CHWs with the older persons and their caregivers, ability of the older persons to answer the AD-8 questions, etc.

### Data analysis

Quantitative data were analysed using Stata version 17.0 (StataCorp LLC, College Station, Texas, United States of America). We summarized continuous variables using means with standard deviations or medians with interquartile ranges and categorical variables using their frequencies and percentages. We conducted the Kappa statistic for agreement between the PCOs and CHWs in screening for cognitive impairment on the AD8. We also compared raw scores of the CHWs to Experts scores using Bland Altman and pair plots and corresponding analyses. The analyses and graphs can be reproduced using Stata’s *diagt*,*roctab* and *concord* commands.

Qualitative data analysis was an iterative process guided by thematic approach and included immersion in the data, identifying meaning units, abstracting content of meaning units and summarizing the importance. Words, sentences or paragraphs that relayed a similar message were grouped as meaning units, which were then condensed and labelled with codes in a code book. Atlas.Ti software was used for the thematic analysis of the data.

## Results

We recruited 385 older persons; 9 persons declined to participate due to lack of time. The median age of the older study participants was 69 years (IQR 64 to 76). The majority (72.2%) of the participants were female, 48.1% had only attained primary school education; 42.6% were married and 41.8% were widowed. The older study participant characteristics are summarised in Table 1. The socio-demographic characteristics of the CHWs are shown in Table 2 below.

**Table 1:** Socio-demographic characteristics of the older persons

Variables	normal (n = 331)	Cognitive impairment (n = 54)	Total (385)	P-value
Age	70.55 (± 9.42)	74.59 (± 11.13)	71.12 (± 9.77)	0.005
Sex			.	0.189
Female	235 (71.00%)	43 (79.63%)	278 (72.21%)	
Male	96 (29.00%)	11 (20.37%)	107 (27.79%)	
Marital status			.	0.362
Married/Co-habiting	146 (44.11%)	18 (33.33%)	164 (42.60%)	
Separated/Divorced	22 (6.65%)	6 (11.11%)	28 (7.27%)	
Single/unmarried	28 (8.46%)	4 (7.41%)	32 (8.31%)	
Widow/widower	135 (40.79%)	26 (48.15%)	161 (41.82%)	
Level of education			.	0.268
No formal education	32 (9.67%)	5 (9.26%)	37 (9.61%)	
Other education	4 (1.21%)	0 (0.00%)	4 (1.04%)	
Primary education or less	153 (46.22%)	32 (59.26%)	185 (48.05%)	
Secondary education	115 (34.74%)	16 (29.63%)	131 (34.03%)	
Tertiary/further education	27 (8.16%)	1 (1.85%)	28 (7.27%)	
What is your current employment status			.	0.191
Housewife/husband	76 (22.96%)	8 (14.81%)	84 (21.82%)	
Paid or self-employment (full time)	20 (6.04%)	2 (3.70%)	22 (5.71%)	
Paid or self-employment (part time)	77 (23.26%)	12 (22.22%)	89 (23.12%)	
Retired due to age - NOT on pension	6 (1.81%)	4 (7.41%)	10 (2.60%)	
Retired due to age-on pension	22 (6.65%)	3 (5.56%)	25 (6.49%)	
Retired due to disability	9 (2.72%)	2 (3.70%)	11 (2.86%)	
Unemployed	113 (34.14%)	23 (42.59%)	136 (35.32%)	
Voluntary employment (unpaid)	8 (2.42%)	0 (0.00%)	8 (2.08%)	

P-values by t-test for continuous variables and Chi2 test for binary/categorical variables.

**Table 2:** Socio-demographic characteristics of the CHWs

Variable	(N = 12) n (%)
**Mean age**	51 SD ± 15
**Sex**	
Female Male	9 (75) 3 (25)
**Marital status**	
Married/co-habiting Separated/divorced Single/unmarried Widow/widower	7 (58.3) 1 (8.3) 2 (16.7) 2 (16.7)
**Level of education**	
Other education Primary education or less Secondary education Tertiary education	1 (8.3) 1 (8.3) 8(66.7) 2 (16.7)
**Employment status**	
Housewife/husband Other Paid/self-employed (full-time) Retired (not on pension) Unemployed Voluntary employment (unpaid)	3 (25) 1 (8.3) 4 (33.4) 1 (8.3) 1 (8.3) 2 (16.7)

The PCOs were 2 males aged 24 and 25 years. They were both single and were diploma holders who had completed their studies the previous year. They were volunteer mental health clinicians at 2 health facilities.

The percentage Kappa agreement between CHWs and research assistants is indicated in Table 3 below.

**Table 3:** Assessment of percentage Kappa agreement between PCOs and CHWs which was calculated at 0.964 agreement

		Examined by PCO		
		Impaired	Normal	Total
Examined by CHWs	Impaired	49	9	58
	Normal	5	322	327
	Total	54	331	385

The Kappa statistic for agreement was **0.964 (Almost perfect agreement)**.

There was 96.4% (CI 94.5–98.2%) agreement between PCOs and CHWs in identifying cognitive impairment with the PCOs identifying 54/385 (14.0: 95%CI 10.7–17.9%) older persons compared to 58/385 (15.1:95% CI 11.6–19.0%) identified by CHWs.

**Of the 58 identified to have cognitive impairment by the CHWs, 93.1% (54/58)** (95% CI 83.3–98.1%) **were referred for further management**.

**Fig. 1:**
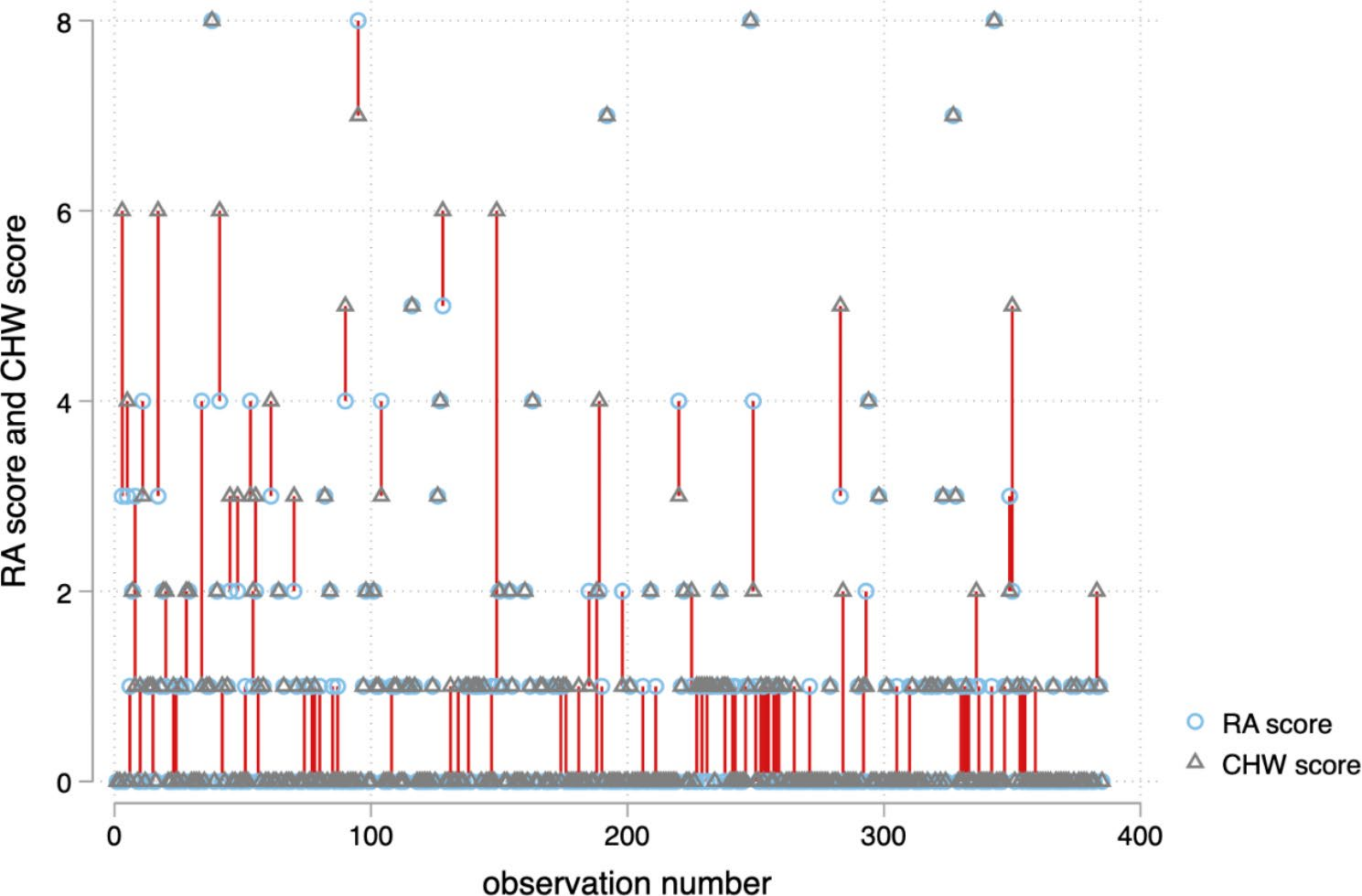
Pair Plot raw showing level of agreement between the PCOs and CHWs scores on the AD8

**Fig. 2:**
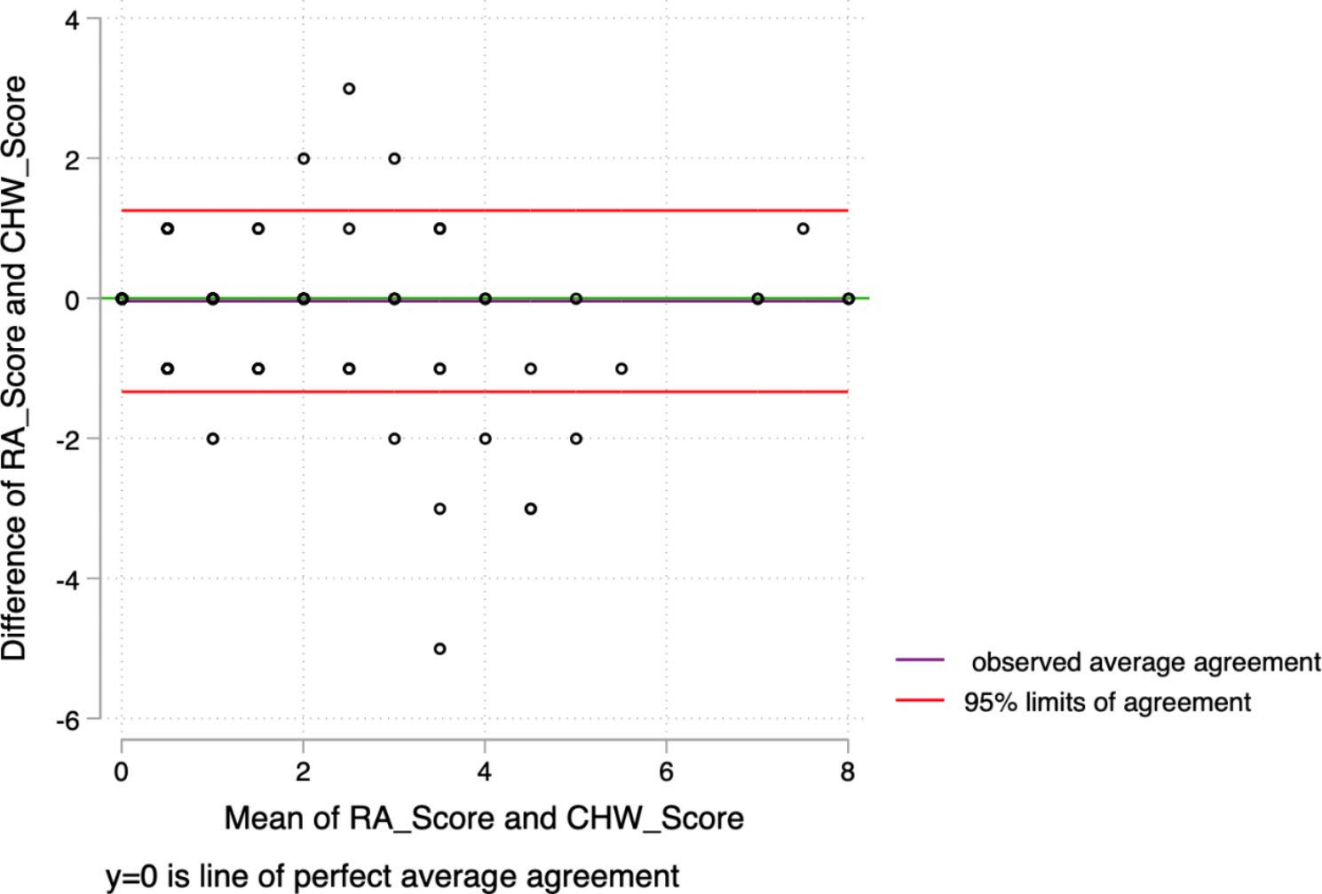
Bland Altman plot. There was an almost perfect agreement between the PCOs and CHWs within the 95% limits of agreement

Corresponding Bland Altman and pair plot showed high agreement between the measurements of CHWs and PCOs (Figs. 1 and 2).

### Qualitative results

We conducted 6 FGDs; 2 for CHWs, 2 for older persons and 2 for the caretakers. Each group had 6–8 participants and thematically analysed them. Their impressions are summarised in the following themes as illustrated by the accompanying quotes of what they said.

#### Theme: feasibility of CHWs in identifying older persons with cognitive impairment

1. Sub-theme: Ease of administration of the screening tool.

The CHWs noted that the AD8 was easy to administer and also easy to understand by the older persons and their caregivers. The older persons were also very welcoming because they had interacted with the CHWs in management of their children and grandchildren in the same homesteads.

*I will say that most of these questions were not hard at our side and even at the side of the older persons because most of the questions we used to ask them in the language they understand and it was easy for them to answer these questions. CHW FGD2*.

*What made this process easy is that we have not been interacting with the older person but since we have been in these communities, they know us very well and that’s why they have been open to us. CHW FGD1*.

*We have been mobilizing them in different ways for example encouraging them to take their children for immunization so they knew us and we didn’t get a lot of difficulties with them. CHW FGD1*.

2. Sub-theme: CHWs were willing to work with older persons.

In addition to working with children and pregnant women, the CHWs were found very willing to work with the older persons.

*I would accept to take care of them if there’s something that is needed in this group of people. I will go and see them one by one so we need to help them because we are also going to age. CHW FGD1*.

*We have been immunizing young children and we also take care of pregnant women so if we add on the aspect of the older persons I think it will empower the CHWs as far as their roles are concerned. CHW FGD2*.

*So this program lifted our level, right you gave us some additional work but this work was exceptional since many older persons liked the program so much and I think we the CHWs are ready to go back to these older persons and extend the service and many are waiting for us to go back. CHW FGD2*.

3. Sub-theme: CHWs as portal of entry.

The CHWs consider themselves as being a portal of entry into the health needs of the community because they work closely with the communities and hence believe they can easily work with the older persons with cognitive impairment.

*I know the CHWs are the people who are there on the ground and I will tell you the people who know the wellbeing of the people in the community are the CHWs and they know much about their people on the ground. CHW FGD2*.

4. Sub-theme: CHWs are respected and trusted by the older persons.

*So, you may see this older person as weak but they still believe in us because of the care we give to the young children that means we can also take care of them. Whatever you advise them they will take it in good faith. CHW FGD1*.

*I think as CHWs we have to take care of the older people because I see it is not fair to visit a home and extend health care services to the children, and pregnant mothers and we forget about the older people needed it is not fair. CHW FGD1*.

5. Sub-theme: CHWs provide psychosocial support for the families.

*We also benefit when these CHWs have finished talking to them, they also train us on how to handle these people. For example, to stop being rude at them, and give them enough care so that they also live a good life. CARETAKERS FGD1*.

*When these CHWs give you some training it helps you to understand these people. They also tell us to take care of them even when they forget the things it always tells us to remind them. CARETAKERS FGD1*.

#### Theme: feasibility of CHWs in referring older persons with cognitive impairment

1. Sub-theme: The CHWs are an important link to care.

CHWs have been known to be the linkage between the community and the health facilities. Some older persons have caretakers that do not clearly understand their mental illness hence the CHWs can reach out to these older persons and their caregivers with knowledge on cognitive impairment and referral for care.

*Yes, we can manage this task … we can do it and we are the bridge between the hospital and the community that means we are able to do it. CHW FGD1*.

*If we the CHWs go back to these older persons and inform them about this study we have been conducted, the health workers have come up with a program of linking them to care they will tell their care-takers to take them to the right hospital for care. CHW FGD2*.

*I think if the program comes and it involves helping the older persons I think they will be able. So if you tell them that we have also a program for the older persons and it is like this if they get a problem they come to you the CHWs or you can link them to the health facility according to what he has told you. OLDER PERSONS FGD1*.

*Yes they can, what I know they may write for them a note and the older person will bring that note to any doctor who will be around. So I think they will do the work and they can’t send these people to the hospital without any information that shows that they have been referred here. CARETAKERS FGD2*.

2. Sub-theme: Following up of referred clients by the CHWs is possible.

The CHWs had referral forms that they give the older persons/ clients to present to the health facility. These referral forms have the reason for referral and a feedback form to be returned to the CHWs for ease of follow up that the client did reach the health facility and receive the intended care.

*So when we get these referrals they are self-explanatory because they indicate why she has been referred here. The health worker receiving this patient will also indicate that he has received the client, so the client has to bring back that letter for easy monitoring and that is what we have been doing. CHW FGD1*.

*They know if we give them the referral letters we can help them when they reach the hospital or to link them to the doctors because they know we usually come to the hospital… CHW FGD1*.

*What I know is that they also do the follow up because when they refer someone to the hospital they will also take some time to come and see if the person they gave a referral letter reached at the hospital. or they can go back and check on these people if they received and took their medications as prescribed. CARETAKERS FGD2*.

3. Sub-theme: Awareness and training needed for the empowerment of the CHWs.

Despite the above evidence for feasibility, the CHWs noted that they had had no knowledge about cognitive issues in older persons and therefore did not pay much attention to their health needs.

*So I think it is high time they are sensitised and made aware of the older persons not to take care of the young children only but also the older persons. CARETAKERS FGD2*.

*Then also we need so many trainings, these trainings will empower when we are doing this work… So if we get these refresher trainings we get more new information that will help the development of new books. CHW FGD2*.

## Discussion

This study set out to assess the feasibility of screening for cognitive impairment and referral of older persons by Community Health Workers (CHWs) in Wakiso district, Uganda. The feasibility was assessed as the percentage of older persons correctly screened and referred by the CHWs for cognitive impairment. We proposed/hypothesized that a percentage above 60% would indicate feasibility. We also sought qualitative views from the participants about the feasibility.

About 72% of the older persons were females. This could be explained by the fact that the number of older females in Wakiso is more than that of males [18]. This could be attributed to the fact that the life expectancy for females in Uganda is 70.1 compared to that for males which is 63.2 [31].

The percentage agreement between CHWs and PCOsin screening for cognitive impairment with the AD8 was 96.4. These findings are in agreement with a study done in Ugandan children which showed that CHWs were 86.6% in agreement with the paediatrician in classifying a fever as malaria or pneumonia [19]. Another study conducted in India showed that it was feasible to train CHWs to screen for oral cancer with a near perfect agreement of (κ = 0.9) between the findings of the community health workers and the dentists [20]. In resource limited settings like Uganda, CHWs have proved to be a useful human resource in management of childhood illnesses like malaria, malnutrition and URTIs [21, 22]. This resource can also be utilised in mental health for older persons within the communities. Studies have also shown that the CHWs are a conduit of health information to their communities hence providing means for early identification of cognitive impairment and other mental health problems among older persons [11, 23].

The CHWs, referred almost all cognitively impaired adults 54 (93.1%) for further management implying that it is feasible for CHWs to refer these older persons after screening them. Other studies that have used CHWs in identifying illnesses in the community have also yielded similar results. A study conducted in Iganga district among children with malaria and pneumonia indicted that CHWs were able to adequately manage those identified with disease 96% of the time [13]. Previous studies have shown that CHWs are an important linkage between far to reach communities and health facilities [11]. A study conducted in KwaZulu-Natal in 2010 among mothers of neonates and infants, indicated that 95% of mothers who were referred by CHWs reached the health facility and accessed care for their babies [24]. The changing family structure in the Ugandan setting [25] calls for employing non family members in the care of older persons and CHWs are a valuable resource that is a port of entry into the health system [26]. However, there is need for continuous training and motivation to facilitate the CHWs as they care for older persons within their communities [21, 27].

Our qualitative results showed that CHWs have a good relationship with the older persons and the communities where they lived and worked. This correlates well with results from previous studies which show that good working relationships between the CHWs and the community promote trust, collaboration and respect among the teams. This further speaks to the feasibility of CHWs in linking the communities to the health facilities [28, 29].

The call for empowerment, training and facilitation of CHWs to enable CHWs carry out screening and referral services in the communities as highlighted in this study is echoed in literature [30, 31].

### Limitations

This was a cross sectional study hence did not comprehensively capture the changes of this feasibility over time.

The AD 8 has not been validated in the Ugandan setting as a screening tool for cognitive impairment.

We selected only the CHWs involved in mobilisation and this could have caused a selection bias.

## Conclusion

We conclude that it is feasible to use CHWs in screening and referring older persons with cognitive impairment for definitive treatment and care. The CHWs are an available resource for linking the older persons to health services; however, they need continuous training and facilitation to carry on this role adequately.

### Recommendation

Further research is needed to replicate the findings of this study and to provide findings on implementation of using the CHWs in the older age-group. This would further strengthen the available scientific evidence on the use of CHWs.

## Electronic supplementary material

Below is the link to the electronic supplementary material.

Supplementary Material 1

## Data Availability

All data generated or analysed during this study are included in this published article [and its supplementary information files].

## List of abbreviations

AD8: Alzheimer’s Disease scale 8
CHWs: Community Health Workers
FGDs: Focus Group Discussions
HIV: Human Immuno-deficiency Virus
IQCODE: Informant questionnaire on cognitive decline in the elderly
P.I: Principal investigator
PCOs: Psychiatric Clinical Officers
RAs: Research assistants
UBACC: University of California, San Diego Brief Assessment of Capacity to Consent
VHTs: Village Health Teams
